# Use of a Large Language Model to Assess Clinical Acuity of Adults in the Emergency Department

**DOI:** 10.1001/jamanetworkopen.2024.8895

**Published:** 2024-05-07

**Authors:** Christopher Y. K. Williams, Travis Zack, Brenda Y. Miao, Madhumita Sushil, Michelle Wang, Aaron E. Kornblith, Atul J. Butte

**Affiliations:** 1 Bakar Computational Health Sciences Institute, University of California, San Francisco; 2 Department of Emergency Medicine, University of California, San Francisco; 3 Department of Pediatrics, University of California, San Francisco

## Abstract

**Importance:**

The introduction of large language models (LLMs), such as Generative Pre-trained Transformer 4 (GPT-4; OpenAI), has generated significant interest in health care, yet studies evaluating their performance in a clinical setting are lacking. Determination of clinical acuity, a measure of a patient’s illness severity and level of required medical attention, is one of the foundational elements of medical reasoning in emergency medicine.

**Objective:**

To determine whether an LLM can accurately assess clinical acuity in the emergency department (ED).

**Design, Setting, and Participants:**

This cross-sectional study identified all adult ED visits from January 1, 2012, to January 17, 2023, at the University of California, San Francisco, with a documented Emergency Severity Index (ESI) acuity level (immediate, emergent, urgent, less urgent, or nonurgent) and with a corresponding ED physician note. A sample of 10 000 pairs of ED visits with nonequivalent ESI scores, balanced for each of the 10 possible pairs of 5 ESI scores, was selected at random.

**Exposure:**

The potential of the LLM to classify acuity levels of patients in the ED based on the ESI across 10 000 patient pairs. Using deidentified clinical text, the LLM was queried to identify the patient with a higher-acuity presentation within each pair based on the patients’ clinical history. An earlier LLM was queried to allow comparison with this model.

**Main Outcomes and Measures:**

Accuracy score was calculated to evaluate the performance of both LLMs across the 10 000-pair sample. A 500-pair subsample was manually classified by a physician reviewer to compare performance between the LLMs and human classification.

**Results:**

From a total of 251 401 adult ED visits, a balanced sample of 10 000 patient pairs was created wherein each pair comprised patients with disparate ESI acuity scores. Across this sample, the LLM correctly inferred the patient with higher acuity for 8940 of 10 000 pairs (accuracy, 0.89 [95% CI, 0.89-0.90]). Performance of the comparator LLM (accuracy, 0.84 [95% CI, 0.83-0.84]) was below that of its successor. Among the 500-pair subsample that was also manually classified, LLM performance (accuracy, 0.88 [95% CI, 0.86-0.91]) was comparable with that of the physician reviewer (accuracy, 0.86 [95% CI, 0.83-0.89]).

**Conclusions and Relevance:**

In this cross-sectional study of 10 000 pairs of ED visits, the LLM accurately identified the patient with higher acuity when given pairs of presenting histories extracted from patients’ first ED documentation. These findings suggest that the integration of an LLM into ED workflows could enhance triage processes while maintaining triage quality and warrants further investigation.

## Key Points

**Question:**

Can a large language model (LLM) accurately assess clinical acuity in the emergency department (ED)?

**Findings:**

This cross-sectional study of 251 401 adult ED visits investigated the potential for an LLM to classify acuity levels of patients in the ED based on the Emergency Severity Index across 10 000 patient pairs. The LLM demonstrated accuracy of 89% and was comparable with human physician classification in a 500-pair subsample.

**Meaning:**

These findings suggest that LLMs could accurately identify higher-acuity patient presentation when given pairs of presenting histories extracted from patients’ first ED documentation.

## Teaser

This cross-sectional study evaluates the ability of a large language model to classify pairs of emergency department (ED) patients presenting histories as higher and lower acuity.

## Introduction

The launch of large language models (LLMs) has generated widespread attention among researchers, the media, and the general public.^1,2^ Recent studies have already suggested high performance on various natural language tasks, including achieving a passing score in the US Medical Licensing Examination and solving publicly available clinical diagnostic challenges such as the *New England Journal of Medicine* clinicopathologic conferences.^3,4^ However, prior research has mainly engaged with simulated health care scenarios not derived from electronic health records, which offer valuable insights but leave an opportunity to evaluate the technology’s application in clinical practice.

As the capabilities of LLMs applied to clinical tasks begin to be investigated, it is important to consider in what areas of medicine these models can be most impactful. Determination of clinical acuity is one of the foundational elements of medical reasoning in emergency medicine and consequently offers an excellent opportunity to explore LLM capabilities using data that are routinely collected during the emergency department (ED) triage process. Emergency department acuity triage, which involves prioritizing patients based on both the severity of their condition and their expected resource needs, ensures that those requiring emergency care are attended to promptly.^5^ This is an inherently challenging task due to the need to quickly estimate patient interventions with only limited information.

The Emergency Severity Index (ESI), a triage scoring system widely used in EDs in the US, uses an algorithm to categorize patients arriving at the ED, estimating the severity of their condition and anticipated future resource use. The ESI is assigned based on a combination of initial vital sign assessments, the patient’s presenting symptoms, and the clinical judgment of the triage clinician, who is often a trained registered nurse. By capturing clinical acuity at triage, the ESI can be used as a surrogate marker to evaluate, at scale, whether LLMs can correctly assess the severity of a patient’s condition on presentation to the ED. This can be achieved by providing the LLM with patient clinical histories documented in ED physician notes, prompting the model to compare histories to determine which patient has the higher acuity, and evaluating the model output against the ground truth as determined by ESI score.

We evaluated the ability of an LLM to classify which patient presentation is the higher acuity, as defined by ESI, within a set of 10 000 ED patient pairs. We hypothesized that the LLM would exhibit comparable performance with the standard of care in evaluating triage acuity severity, potentially affecting future ED clinical workflows and quality improvement initiatives.

## Methods

In this cross-sectional study, we identified all adult (aged ≥18 years) visits to the University of California, San Francisco (UCSF), ED from January 1, 2012, to January 17, 2023, with a documented ESI acuity level (immediate [highest], emergent, urgent, less urgent, and nonurgent [lowest]) and with a corresponding ED physician note created during the encounter. These clinical notes have been deidentified, externally certified, and made available for research as previously described.^6^ The UCSF Institutional Review Board determined that this use of deidentified structured and clinical text data in the UCSF Information Commons is not considered human participant research and that the study was therefore exempt from further approval and the need for informed consent. This study followed the Strengthening the Reporting of Observational Studies in Epidemiology (STROBE) reporting guideline.

Emergency department visits with no associated clinical notes were excluded, as were visits without clinical notes written by emergency medicine clinicians. If more than 1 ED clinician note was available for a particular ED visit, the earliest note was selected. In the case of multiple notes with the same time, the longest note (by word count) was selected. From this corpus of deidentified clinical text, software was written using regular expressions to extract the chief concern, history of presenting illness, and review of systems sections from each note that constitute a patient’s clinical history (eMethods in Supplement 1). We chose to include only patients’ clinical history into the LLM queries since, in contrast to vital signs and other examination findings, this information may be obtained in the future in an automated manner without the requirement for clinician physical examination.

The ESI is the triage system recommended by the American College of Emergency Physicians and Emergency Nurses Association.^7^ It is recorded during the initial triage of patients on presentation to the ED and provides an indication of how acutely unwell a patient is, how urgently they require medical attention, and the number of anticipated resources required during their encounter. Scoring of the ESI is performed by clinical staff following an established protocol that incorporates information readily available at triage, including a patient’s presenting concerns, the presence or absence of high-risk presenting symptoms, and triage vital signs.^7^ There are 5 acuity levels based on how urgently patients need to be seen by the physician or other clinician: immediate, emergent, urgent, less urgent, and nonurgent.^7^ In this study, the ESI was used as the ground-truth indication of which patient presented with a higher clinical acuity, allowing a comparison between the LLMs and human (resident physician) inference.

We randomly selected, with replacement, a sample of 10 000 pairs of ED visits with nonequivalent ESI scores, balanced for each of the 10 possible pairs of 5 ESI scores. Accessing the application programming interface via the Health Insurance Portability and Accountability Act–compliant UCSF Secure Azure OpenAI environment, we queried GPT-4 (OpenAI model = “gpt-4-0314”; role = “user”; temperature = 0; all other settings at default values) to consider the clinical history of each pair of ED presentations and return which patient had a higher-acuity presentation. We prompted the LLM to return only 1 of 2 options: “Patient A is of higher acuity” or “Patient B is of higher acuity” (full details are provided in eMethods 1 in Supplement 1) in a zero-shot manner (where each pair of ED presentations was considered independently of other pairs), with no additional in-context learning or fine-tuning. The patient with the higher-acuity presentation (A or B) was extracted from the application programming interface output using regular expressions and compared with the ground-truth acuity level. We additionally queried GPT-3.5 Turbo (OpenAI model = “gpt-3.5-turbo-0301”; role = “user”; temperature = 0; all other settings at default values) to allow comparison between versions. A balanced 500-pair subsample (50 for each of the 10 categories) was manually classified by a resident physician with 2 years of postgraduate general medical training (C.Y.K.W.) for comparison of the performance between the LLM models and human classification. In addition, an attending emergency medicine physician (A.E.K.) independently classified 10% of this subsample, with 90% agreement between reviewers.

### Statistical Analysis

Accuracy scores with bootstrapped 95% CIs were calculated for both LLMs and the human annotator for comparison. We additionally calculated a weighted accuracy score by multiplying the individual scores in each of the 10 categories by the proportional distribution of paired ESI categories from the original, unbalanced, cohort of 251 401 ED visits (eFigure and eTable 1 in Supplement 1). All analyses were conducted in Python, version 3.11 (Python Software Foundation). Nonoverlapping CIs determined statistical significance.

## Results

**Figure 1.**
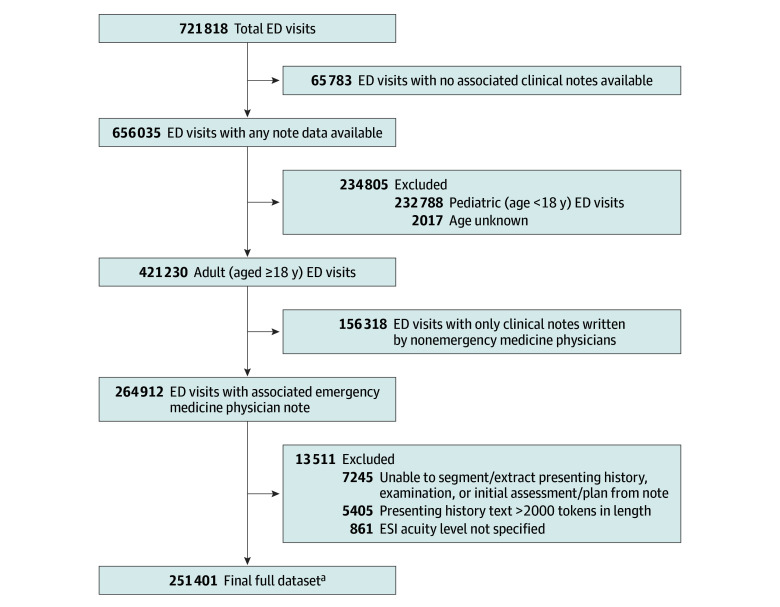
Flowchart of Included Emergency Department (ED) Visits ESI indicates Emergency Severity Index (immediate, emergent, urgent, less urgent, and nonurgent). ^a^A balanced sample of 10 000 patient pairs was created from the full sample wherein each pair comprised patients with the following disparate ESI acuity scores: immediate/emergent (n = 1000); immediate/urgent (n = 1000); immediate/less urgent (n = 1000); immediate/nonurgent (n = 1000); emergent/urgent (n = 1000); emergent/less urgent (n = 1000); emergent/nonurgent (n = 1000); urgent/less urgent (n = 1000); urgent/nonurgent (n = 1000); less urgent/nonurgent (n = 1000).

From a total of 251 401 adult ED visits, we created a balanced sample of 10 000 patient pairs wherein each pair comprised patients with disparate ESI acuity scores (Figure 1). Using only the information documented in the clinical history sections of patients’ first ED physician notes, we queried the LLM to identify the patient with the highest acuity in each pair. Across this sample of paired patient histories, the LLM correctly inferred the higher acuity patient for 8940 of 10 000 pairs, with an accuracy of 0.89 (95% CI, 0.89-0.90) (eTable 2 in Supplement 1). As expected, model performance improved as ED triage acuity scores became more extreme between pairs (Table 1), with up to 100% accuracy when distinguishing patients with immediate from nonurgent acuity levels. When calculating mean LLM performance weighted according to the natural distribution of ESI pairs in our original cohort, there was a small reduction in overall accuracy to 83% (eTable 1 in Supplement 1).

**Table 1.** Evaluation of Large Language Model Accuracy for Each Type of ESI Acuity Level Pairing Across 10 000 Patient Pairs

Acuity level	ESI acuity level, accuracy (95% CI)^a^				
	Immediate	Emergent	Urgent	Less urgent	Nonurgent
Immediate	NA	NA	NA	NA	NA
Emergent	0.86 (0.84-0.88)	NA	NA	NA	NA
Urgent	0.95 (0.94-0.96)	0.75 (0.72-0.78)	NA	NA	NA
Less urgent	0.99 (0.99-1.00)	0.95 (0.94-0.97)	0.85 (0.83-0.87)	NA	NA
Nonurgent	1.00 (0.99-1.00)	0.98 (0.97-0.99)	0.92 (0.90-0.93)	0.68 (0.66-0.71)	NA

Abbreviations: ESI, Emergency Severity Index; NA, not applicable.

^a^ Overall accuracy was 0.89 (95% CI, 0.89-0.90).

**Figure 2.**
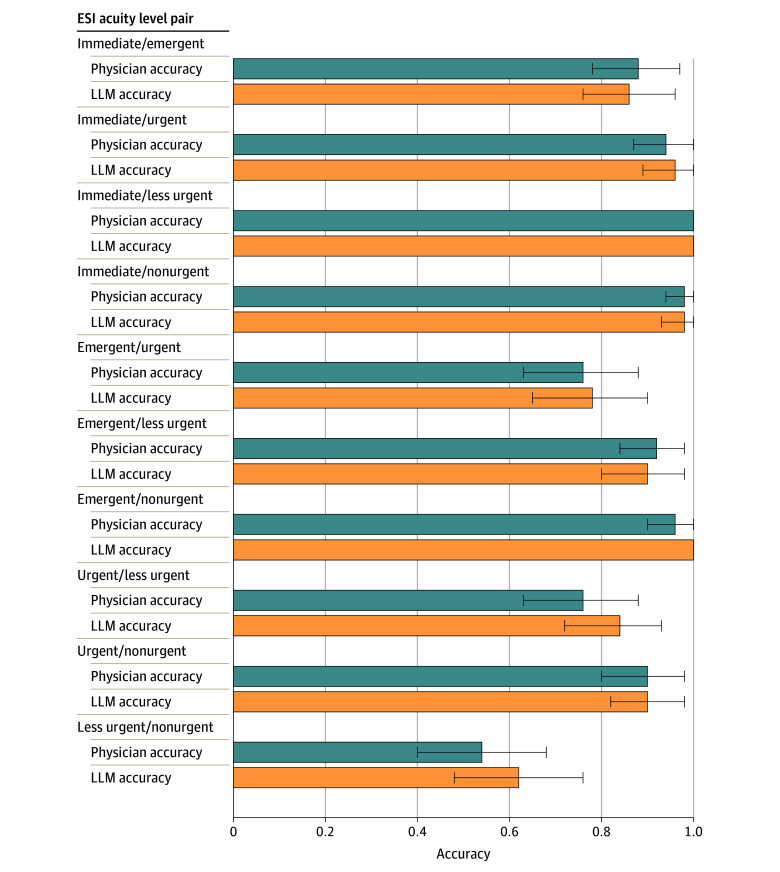
Comparison of Large Language Model (LLM) and Physician Performance Evaluated for each type of Emergency Severity Index (ESI) acuity level pairing in the 500-pair subsample (immediate, emergent, urgent, less urgent, and nonurgent). Overall LLM accuracy was 0.88 (95% CI, 0.86-0.91); overall physician accuracy, 0.86 (95% CI, 0.83-0.89). Error bars indicate 95% CIs.

**Figure 3.**
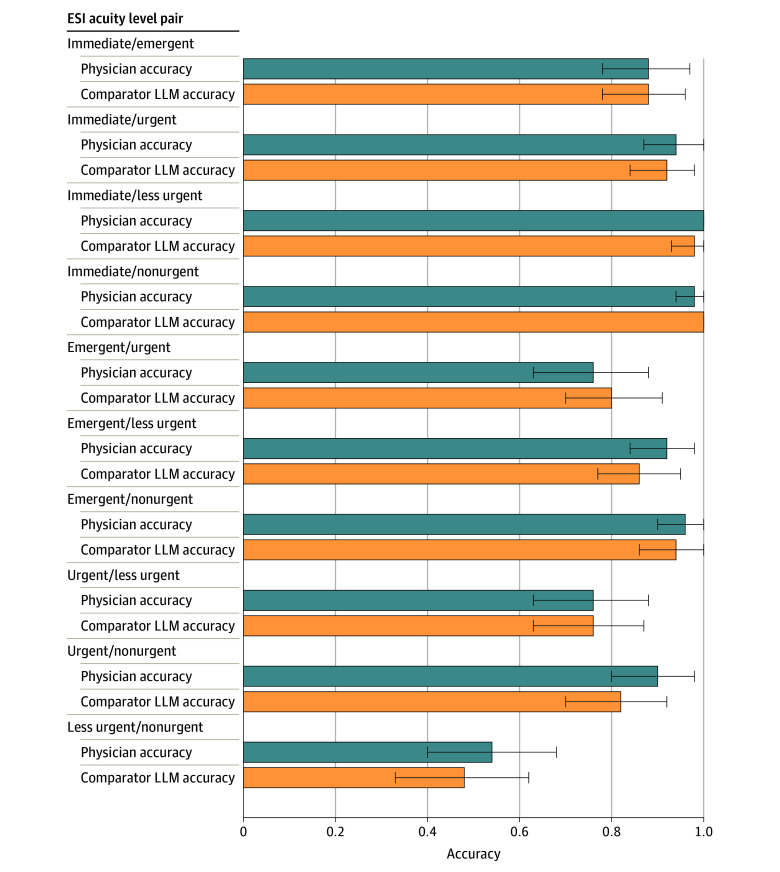
Comparison of Comparator Large Language Model (LLM) and Physician Performance Evaluated for each type of Emergency Severity Index (ESI) acuity level pairing in the 500-pair subsample (immediate, emergent, urgent, less urgent, and nonurgent). Overall comparator LLM accuracy was 0.84 (95% CI, 0.81-0.88); overall physician accuracy, 0.86 (95% CI, 0.83-0.89). Error bars indicate 95% CIs.

Among the 500-pair subsample that was also manually classified, LLM performance (accuracy, 0.88 [95% CI, 0.86-0.91]) was comparable to that of the resident physician (accuracy, 0.86 [95% CI, 0.83-0.89]) (Figure 2 and eTable 3 in Supplement 1), again using only the clinical history sections of the ED physician note. Performance of the comparator LLM was below that of the LLM being studied, with an accuracy of 0.84 (95% CI, 0.83-0.84) (Table 2 and Figure 3).

**Table 2.** Evaluation of Comparator Large Language Model Accuracy for Each Type of ESI Acuity Level Pairing Across 10 000 Patient Pairs

Acuity level	ESI acuity level, accuracy (95% CI)^a^				
	Immediate	Emergent	Urgent	Less urgent	Nonurgent
Immediate	NA	NA	NA	NA	NA
Emergent	0.83 (0.81-0.85)	NA	NA	NA	NA
Urgent	0.93 (0.91-0.95)	0.71 (0.68-0.74)	NA	NA	NA
Less urgent	0.98 (0.97-0.99)	0.88 (0.86-0.90)	0.74 (0.71-0.77)	NA	NA
Nonurgent	0.98 (0.97-0.99)	0.92 (0.90-0.93)	0.81 (0.79-0.83)	0.58 (0.55-0.61)	NA

Abbreviations: ESI, Emergency Severity Index; NA, not applicable.

^a^ Overall accuracy was 0.84 (95% CI 0.83-0.84).

## Discussion

This study represents an early and highly powered evaluation of the ability of an LLM to assess clinical text and stratify patients in the ED based on clinical acuity. We found that the LLM could accurately identify the patient with the higher-acuity presentation when given pairs of presenting histories extracted from patients’ first ED documentation. Among the subsample of patient pairs assessed by both the LLM and physician, LLM performance was comparable with that of the resident physician.

Our findings suggest that the incorporation of LLMs into the ED clinical workflow could offer a significant opportunity to provide triage acuity assignments that are on par with existing practices. Overall, the LLM’s only significant performance weakness was in distinguishing patients assigned a less urgent vs nonurgent acuity, which is unlikely to have significant clinical consequences. In addition, this performance was achieved despite providing only patients’ clinical history to the LLM, omitting the vital signs and other physical examination findings that may be available to triage clinicians on initial evaluation. We intentionally provided the LLMs with patients’ clinical history only because often the availability of the triage clinician, who is needed to obtain vital sign measurements and a brief physical examination of the patient, is the rate-limiting step in ED triage.

The performance of the LLM being studied was superior to that of the comparator LLM, with an accuracy of 89% compared with 84% across the 10 000-pair sample. This is in keeping with previous literature^2,8,9^ that has noted significant differences in performance between these LLMs across both clinical and general natural language processing tasks. However, since details of model training and architecture are not publicly available, it is difficult to assess the reasons for this performance difference.

Prior investigation of the capabilities of LLMs in medicine has largely been confined to curated medical case challenges, including the *New England Journal of Medicine* clinicopathologic conference series, question-answer datasets such as for the US Medical Licensing Examination, and other clinical natural language processing benchmarks, with limited evaluation of their performance when applied to clinical notes.^3,4,10^ More recently, emerging studies have shown strong performance of LLMs when applied to clinical information extraction tasks, such as phenotyping patients with postpartum hemorrhage or labeling breast cancer pathology reports.^11,12^ In this study, we sought to explore the more complex problem of clinical evaluation: namely, can LLMs assimilate information about different types of clinical presentation, weigh the relative severity of each presentation, and decide which of 2 patients is more acutely unwell?

This is perhaps a simpler task compared with prompting the LLM to provide a formal ESI score for each patient. However, LLMs have been trained specifically to follow instructions.^13^ Because of the algorithmic nature of the ESI scoring protocol, it is possible that the LLMs may in fact perform better at this alternative task than they do in the current side-by-side comparison of patients. Moreover, the current task is arguably closer to the reality of clinical practice where identifying, for example, which patient you would give your last resuscitation bed to is more important than knowing what absolute ESI score any given patient has. A notable omission from this task is a comparison of 2 patients with equivalent ESI score; it is unclear how the LLM might consider such a situation, when the most appropriate response may be “neither patient.” Future work should not only examine this further, but also seek to better understand how LLMs work toward assigning a higher acuity to any particular patient and assess LLM performance against more clinically relevant outcomes that incorporate resource utilization, admission status, and mortality status alongside ESI score.

This study highlights the potential application of LLMs to streamline the triage process by identifying patients who are more acutely unwell than others. This is particularly noteworthy given that the LLMs we investigated are general-purpose models that are not specifically fine-tuned for the medical domain. It remains unclear whether domain-specific, medical language models may outperform these state-of-the-art general-purpose LLMs at different medical tasks,^14,15^ though one model that has been fine-tuned for medical question answering has shown promising results.^10,16^ We propose further investigation into the potential implementation of LLMs within the ED triage workflow before they are deployed in clinical practice.

### Limitations

This study has several limitations. First, there may be a time delay between a patient’s initial triage and their review by the ED physician. Consequently, there may be more information available in the ED physician note, used by the LLMs to determine which patient had the higher-acuity presentation in each pair than was available when the ESI acuity score was originally assigned. This reflects the dynamic nature of a patient’s clinical status and acuity, which may change over their ED stay. We therefore examined how reflective a patient’s initial ESI score is of their subsequent clinical outcome across our sample of 10 000 patient pairs (eTable 4 in Supplement 1), demonstrating that patients with higher-acuity presentation were more likely to both be admitted to the hospital and die within 30 days of hospital admission. Although we have used the ESI score as our surrogate marker for ground-truth patient acuity, it is traditionally intended to reflect both the acuity of the presentation and the patient’s anticipated resource needs, which may not always be interrelated.^7^ For certain presentations, patients may receive ESI scores that do not wholly reflect their clinical acuity if they have higher- or lower-than-expected resource needs associated with their presentation type. Second, we did not perform additional prompt engineering to further optimize LLM performance; further iterations of prompt engineering and/or the incorporation of in-context learning could lead to improved LLM performance.^16^ Third, due to the pairwise nature of our sample, it is not possible to reliably calculate model performance across different patient characteristics such as gender and race and ethnicity. Future studies should seek to explicitly test LLM performance for different sociodemographic characteristics across a range of medical tasks to ensure equity among all patient populations. Fourth, we acknowledge that certain elements of the clinical history, such as the history of presenting illness and review of systems, are currently obtained by medical professionals, limiting the present use of this system in early ED triage. However, we believe this will change as LLMs become more integrated within clinical practice, especially given recent reports on the deployment of ambient artificial intelligence scribes at a large health care system.^17^ Last, few details about training for the LLMs we investigated have been released by the model’s manufacturer, which may perpetuate algorithmic bias.^18,19^

## Conclusions

In this cross-sectional study of 10 000 pairs of ED visits, an LLM accurately identified the patient with higher acuity when given pairs of presenting histories extracted from patients’ first ED documentation. Our findings suggest that an LLM could perform the complex task of evaluating clinical acuity. The integration of LLMs into ED workflows could enhance triage processes while maintaining triage quality and warrants further investigation.

## Notes

Supplement 1. **eMethods.** Study Sample **eFigure.** Distribution of Emergency Severity Index (ESI) Acuity Levels in the Original (N = 251 041) Cohort of ED Visits **eTable 1.** Calculation of Weighted Average GPT-3.5 Turbo and GPT-4 Accuracy **eTable 2.** Confusion Matrix for Which Patient Had the Higher ESI Acuity Score Among the Balanced Sample of 10 000 Patient Pairs **eTable 3.** Confusion Matrix for Which Patient Had the Higher ESI Acuity Score Among the Balanced Subsample of 500 Patient Pairs **eTable 4.** Hospital Admission and 30-Day Mortality Rate Among Patients With Different ESI Scores in Our 10 000 Pair Sample
eReference

Supplement 2.
Data Sharing Statement
