# Supplementary material for: Use of a Large Language Model to Assess Clinical Acuity of Adults in the Emergency Department
Source: JAMA Netw Open. 2024 May 7;7(5):e248895. doi: 10.1001/jamanetworkopen.2024.8895 (PMC11077390; doi:10.1001/jamanetworkopen.2024.8895)
Supplement: Supplement 1. — eMethods. Study Sample eFigure. Distribution of Emergency Severity Index (ESI) Acuity Levels in the Original (N = 251 041) Cohort of ED Visits eTable 1. Calculation of Weighted Average GPT-3.5 Turbo and GPT-4 Accuracy eTable 2. Confusion Matrix for Which Patient Had the Higher ESI Acuity Score Among the Balanced Sample of 10 000 Patient Pairs eTable 3. Confusion Matrix for Which Patient Had the Higher ESI Acuity Score Among the Balanced Subsample of 500 Patient Pairs eTable 4. Hospital Admission and 30-Day Mortality Rate Among Patients With Different ESI Scores in Our 10 000 Pair Sample eReference [file jamanetwopen-e248895-s001.pdf]

Supplementary Online Content

Williams CYK, Zack T, Miao BY, et al. Use of a large language model to assess clinical acuity of adults in the emergency department. *JAMA Netw Open*. 2024;7(5):e248895. doi:10.1001/jamanetworkopen.2024.8895

eMethods. Study Sample

eFigure. Distribution of Emergency Severity Index (ESI) Acuity Levels in the Original (N = 251 041) Cohort of ED Visits

eTable 1. Calculation of Weighted Average GPT-3.5 Turbo and GPT-4 Accuracy

eTable 2. Confusion Matrix for Which Patient Had the Higher ESI Acuity Score Among the Balanced Sample of 10 000 Patient Pairs

eTable 3. Confusion Matrix for Which Patient Had the Higher ESI Acuity Score Among the Balanced Subsample of 500 Patient Pairs

eTable 4. Hospital Admission and 30-Day Mortality Rate Among Patients With Different ESI Scores in Our 10 000 Pair Sample

eReference

This supplementary material has been provided by the authors to give readers additional information about their work.

© 2024 Williams CYK et al. *JAMA Network Open*.

eMethods. Study Sample

Note pre-processing & segmentation

Clinical notes were minimally preprocessed - only new lines and extra spaces were removed. A series of Regular Expressions were used to examine the structure of notes, confirming the presence/absence of the following note headers: 'Chief Complaint' (261,688/264,912 notes); 'Review of Systems' (261,554/264,912 notes); 'Physical Exam' (263,702/264,912 notes); 'ED Course' (232,778/264,912 notes); and 'Initial Assessment' (186,620/264,912 notes). For each clinical note, we extracted all text from:

1) Clinical History: section 'Chief Complaint' (inclusive) to 'Physical Exam', representing the full history of each patient's ED visit, including both their Presenting Complaint/History of Presenting Complaint and Systems Review;

2) Examination: section 'Physical Exam' (inclusive) to either 'ED course' or 'Initial Assessment', representing the Physical Examination findings; and

3) Assessment/Plan: from 'ED course' or 'Initial Assessment' to note end, representing the clinician's Impression/Assessment and Plan.

Tokenisation

A sample of the segmented note text was examined to confirm proper extraction. The dataset was subsequently filtered to remove ED visits with an unspecified ESI acuity score. Only ED visits in which all three sections of the accompanying Emergency Medicine Provider note could be segmented and extracted were included. For this study, only text from the Clinical History section of patients' clinical notes was analysed by GPT-4/GPT-3.5-turbo.

The number of tokens for each section was calculated using the *tiktoken* tokenizer module recommended by Open AI. Tokens can be thought of as pieces of words which form the input of large language models; 100 tokens are approximately equal to 75 words.[1] Notably, GPT-3.5-turbo has a maximum limit of 4096 tokens shared between prompt (input) and completion (output). Because our prompt required a comparison of Clinical Histories between two different patients presenting to the ED, we further filtered our dataset to remove the minority of ED visits with a Clinical History of greater than 2000 tokens in length.

© 2024 Williams CYK et al. *JAMA Network Open*.

Sample selection

Following the creation of this master dataset, we selected, with replacement, a 10,000 pair sample on which GPT-4/GPT-3.5-turbo performance was evaluated. This sample was balanced for each of the 10 paired classes of ESI acuity score:

- 1000 'Immediate' : 'Emergent' pairs of ED visits
- 1000 'Immediate' : 'Urgent' pairs of ED visits
- 1000 'Immediate' : 'Less Urgent' pairs of ED visits
- 1000 'Immediate' : 'Non-Urgent' pairs of ED visits
- 1000 'Emergent' : 'Urgent' pairs of ED visits
- 1000 'Emergent' : 'Less Urgent' pairs of ED visits
- 1000 'Emergent' : 'Non-Urgent' pairs of ED visits
- 1000 'Urgent' : 'Less Urgent' pairs of ED visits
- 1000 'Urgent' : 'Non-Urgent' pairs of ED visits
- 1000 'Less Urgent' : 'Non-Urgent' pairs of ED visits

This sample size was chosen to reflect an appropriate balance between selecting a sufficiently diverse range of patient presentations and the increasing cost of model inference. Similarly, the n=500 sample for manual annotation was considered large enough (at 5% of the original sample) to allow evaluation of model performance across each of the 10 paired ESI combinations, but small enough to allow completion of the time-intensive manual annotation process within a reasonable timeframe.

GPT-4/GPT-3.5-turbo prompt

We used GPT-4/GPT-3.5-turbo to perform zero shot classification of which patient was of a higher acuity based on their Clinical History. Using Regular Expressions, we confirmed that there was no mention of a patient's acuity level in their Clinical History to ensure no data leakage would confound our results. We deployed the following template for prompting GPT-4/GPT-3.5-turbo, with Patient A and Patient B representing the two Clinical Histories for any particular pair of ED visits:

You are an Emergency Department physician. Below are the symptoms of two different patients presenting to the Emergency Department, Patient A and Patient B. Please return which patient is of the highest acuity between these two patients. Please return one of two answers: '0: Patient A is of higher acuity' '1: Patient B is of higher acuity' Please do not return any additional explanation.

Patient A: " "

Patient B: " "

© 2024 Williams CYK et al. *JAMA Network Open*.

This template was chosen following several rounds of prompt engineering to ensure that only the two stated outputs ('0: Patient A is of higher acuity' or '1: Patient B is of higher acuity') were returned by the model. This was necessary as GPT-4/GPT-3.5-turbo has a tendency to return verbose answers which otherwise would be difficult to analyse at scale. We did not conduct additional prompt engineering to further improve model performance.

We randomly shuffled whether patient A or B was the higher acuity patient to prevent possible systemic bias in the way GPT-4/GPT-3.5-turbo returns a response from confounding our results (e.g if GPT-4/GPT-3.5-turbo is more likely to return ‘Patient A’ as its response, regardless of the Clinical History given).

© 2024 Williams CYK et al. *JAMA Network Open*.

eFigure. Distribution of Emergency Severity Index (ESI) Acuity Levels in the Original (N = 251 041) Cohort of ED Visits

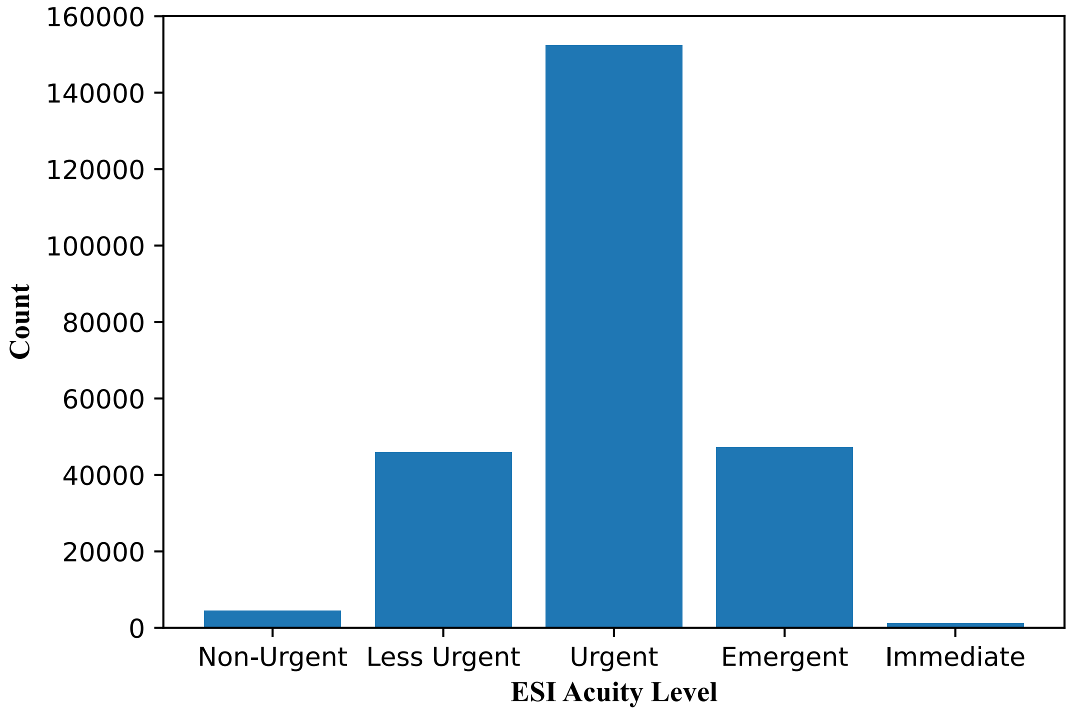

© 2024 Williams CYK et al. *JAMA Network Open*.

eTable 1. Calculation of Weighted Average GPT-3.5 Turbo and GPT-4 Accuracy

**Acuity Pair**	**Original cohort distribution (no. of pairs)**	**Weight**	**GPT-3.5-turbo accuracy (unweighted)**	**GPT-3.5-turbo accuracy (weighted)**	**GPT-4 accuracy (unweighted)**	**GPT-4 accuracy (weighted)**
Immediate – Emergent	47232 x 1200 = 56678400	0.003	0.83	0.002	0.86	0.003
Immediate – Urgent	152437 x 1200 = 182924400	0.01	0.93	0.009	0.95	0.01
Immediate – Less Urgent	45995 x 1200 = 55194000	0.003	0.98	0.003	0.99	0.003
Immediate – Non-Urgent	4537 x 1200 = 5444400	0.000	0.98	0.000	1.00	0.000
Emergent – Urgent	152437 x 47232 = 7199904384	0.405	0.71	0.288	0.75	0.304
Emergent – Less Urgent	45995 x 47232 = 2172435840	0.122	0.88	0.107	0.95	0.116
Emergent – Non-Urgent	4537 x 47232 = 214291584	0.012	0.92	0.011	0.98	0.012
Urgent – Less Urgent	45995 x 152437 = 7011339815	0.394	0.74	0.292	0.85	0.335
Urgent – Non-Urgen	4537 x 152437 = 691606669	0.039	0.81	0.032	0.92	0.036
Less Urgent – Non-Urgent	4537 x 45995 = 208679315	0.012	0.58	0.007	0.68	0.008
			Weighted	**0.75**		**0.83**

© 2024 Williams CYK et al. *JAMA Network Open*.

			average accuracy			

Weighted according to the relative distribution of each acuity class pair in the original (n = 251,041) cohort of ED visits.

eTable 2. Confusion Matrix for Which Patient Had the Higher ESI Acuity Score Among the Balanced Sample of 10 000 Patient Pairs

**Higher acuity patient ('A' or 'B')**	a) GPT-4		b) GPT-3.5-turbo	
	GPT-4 label: A	GPT-4 label: B	GPT-3.5-turbo label: A	GPT-3.5-turbo label: B
Ground-truth label: A	*4313*	*687*	*4016*	*984*
Ground-truth label: B	*373*	*4627*	*662*	*4338*

a) GPT-4 labels and b) GPT-3.5-turbo labels compared to ground-truth (extracted from the electronic health record). Overall GPT-4 accuracy = 8940/10000 = 0.89. Overall GPT-3.5-turbo accuracy = 8354/10000 = 0.84.

eTable 3. Confusion Matrix for Which Patient Had the Higher ESI Acuity Score Among the Balanced Subsample of 500 Patient Pairs

**Higher acuity patient ('A' or 'B')**	a) GPT-4		b) GPT-3.5-turbo		c) Resident physician	
	GPT-4 label: A	GPT-4 label: B	GPT-3.5-turbo label: A	GPT-3.5-turbo label: B	Physician label: A	Physician label: B
Ground-truth label: A	*236*	*38*	*220*	*54*	*234*	*40*
Ground-truth label: B	*20*	*206*	*24*	*202*	*28*	*198*

a) GPT-4 labels, b) GPT-3.5-turbo labels and c) resident physician labels compared to ground-truth (extracted from the electronic health record). GPT-4 accuracy = 442/500 = 0.88; GPT-3.5-turbo accuracy = 422/500 = 0.84; resident physician accuracy = 432/500 = 0.86.

eTable 4. Hospital Admission and 30-Day Mortality Rate Among Patients With Different ESI Scores in Our 10 000 Pair Sample

**Acuity level**	**Admitted to hospital from ED, n (%)**	**Died within 30 days of ED presentation, n (%)**
Immediate	2835 (70.9%)	1530 (38.3%)
Emergent	1919 (48%)	161 (4%)
Urgent	891 (22.3%)	35 (0.9%)
Less Urgent	84 (2.1%)	2 (0.1%)
Non-Urgent	41 (1.0%)	4 (0.1%)

© 2024 Williams CYK et al. *JAMA Network Open*.

eReference

1. What are tokens and how to count them? | OpenAI Help Center. Accessed March 29, 2023. https://help.openai.com/en/articles/4936856-what-are-tokens-and-how-to-count-them

© 2024 Williams CYK et al. *JAMA Network Open*.
