# Supplementary material for: Use of a Large Language Model to Assess Clinical Acuity of Adults in the Emergency Department
Source: JAMA Netw Open. 2024 May 7;7(5):e248895. doi: 10.1001/jamanetworkopen.2024.8895 (PMC11077390; doi:10.1001/jamanetworkopen.2024.8895)
Supplement: Supplement 2. — Data Sharing Statement [file jamanetwopen-e248895-s002.pdf]

Data Sharing Statement

Williams. Use of a Large Language Model to Assess Clinical Acuity of Adults in the Emergency Department. *JAMA Netw Open*. Published May 07, 2024.
doi:10.1001/jamanetworkopen.2024.8895

Data
Data available: No
